# The Effect of Leucine-Enriched β-Lactoglobulin Versus an Isonitrogenous Whey Protein Isolate on Skeletal Muscle Protein Anabolism in Young Healthy Males

**DOI:** 10.3390/nu17213410

**Published:** 2025-10-30

**Authors:** Isabel A. Ely, Melanie Paul, Joshua J. S. Wall, Jake Cox, Mads S. Larsen, Paula J. Scaife, Jon N. Lund, Leigh Breen, Daniel J. Wilkinson, Kenneth Smith, Bethan E. Phillips, Philip J. Atherton

**Affiliations:** 1 Centre of Metabolism Ageing & Physiology, School of Medicine, Nottingham NIHR Biomedical Research Centre, MRC-Versus Arthritis Centre for Musculoskeletal Ageing Research (CMAR), University of Nottingham, Royal Derby Hospital, Derby DE22 3DT, UKmbyjc16@exmail.nottingham.ac.uk (M.P.); joshua.wall@nhs.net (J.J.S.W.); mbyjc16@nottingham.ac.uk (J.C.);; 2 Department of Surgery, Royal Derby Hospital, Derby DE22 3NE, UK; 3 Arla Foods Ingredients Group P/S, 8260 Viby J, Denmark; 4 Diabetes Research Centre, College of Life Sciences, University of Leicester, Leicester LE5 4PW, UK; 5 Department of Sport and Health Sciences, Ritsumeikan University, Kyoto 603-8577, Japan

**Keywords:** skeletal muscle, leucine, protein, exercise

## Abstract

Background: β-lactoglobulin (BLG) is a protein found within whey protein (WP) that is rich in essential amino acids, most notably, leucine (LEU). LEU is considered the most potent EAA in the postprandial stimulation of muscle protein synthesis (MPS), such that suboptimal protein/essential amino acid (EAA) doses containing higher LEU content elicit muscle anabolism comparable to larger protein doses. Our objective was to test the effects of naturally LEU-rich BLG (~10 g protein) versus isonitrogenous whey protein isolate (WPI, ~10 g) on MPS. Methods: Ten healthy young men (26 ± 2 y; 179 ± 2 cm; 81 ± 3 kg) received BLG (1.57 g LEU) or WPI (1.02 g LEU) in a randomised double-blind cross-over fashion. A primed constant intravenous infusion of [1,2 ^13^C_2_] LEU was used to determine MPS (isotope ratio mass spectrometry) at baseline and in response to feeding (FED) and feeding-plus-exercise (FED-EX; 6 × 8 unilateral leg extensions; 75% 1-RM). Plasma insulin and EAA’s were quantified. Results: Plasma EAA, branched-chain amino acid (BCAA), and LEU concentrations increased rapidly following both protein supplements but exhibited a significantly greater EAA/BCAA/leucinemia following BLG (*p* < 0.05 for all). MPS increased significantly in both FED (~52%) and FED-EX (~58%) states, with no significant differences between supplements. Conclusions: Both BLG and WPI effectively stimulated MPS doses in young healthy males, with BLG offering an advantage in EAA/BCAA/LEU bioavailability. It follows that future research should explore the potential of BLG in populations exhibiting anabolic resistance and exercise anabolism deficiency, such as older adults as well as frail and clinical populations, to assess its utility in preserving muscle mass under conditions of suboptimal protein intake.

## 1. Introduction

Bovine milk protein consists of two major fractions: whey (~20%) and micellar casein (~80%). Both protein fractions are among the highest-quality sources of dietary protein based on indexes of protein quality [1,2,3,4,5] yet distinct plasma amino acid (AA) profiles are observed following consumption. Specifically, ingestion of whey protein (WP) results in rapid and substantial increases in postprandial plasma essential amino acids (EAAs) including leucine (LEU), thus being characterised as a rapidly digested protein [6]. In contrast, micellar casein coagulates and precipitates in the low-pH environment of the stomach following ingestion [7]. This results in casein exhibiting a slower, protracted aminoacidaemia [6], though that is not to say a more protracted, lesser aminoacidaemia may be of benefit in some instances. Previous research has highlighted muscle protein synthesis (MPS) responses to be superior following WP consumption [8,9] or show no differences compared to other ‘high-quality’ (e.g., casein) protein sources [10,11,12]. Such apparent conflicting results may be influenced by adjuvant exercise in the protocol, feeding strategy (i.e., pulse/bolus), or the timing of skeletal muscle (SKM) biopsies for the quantification of MPS.

β-lactoglobulin (BLG) is a protein found within WP and accounts for 45–57% of bovine whey proteins [13,14,15]. BLG is a protein source naturally abundant in EAA, especially LEU, such that the LEU content of BLG exceeds the constituent LEU content of WP by ~50% (~15% vs. ~10%) [14]. Crucially, LEU is the most potent EAA for stimulating MPS [16,17] and a key nutrient regulator of translation initiation enacting the postprandial acceleration of MPS [18]. Subsequently, there has been increasing interest in the role of LEU in enhancing SKM anabolism, and ultimately increasing and/or maintaining SKM mass, especially in the context of ageing, exercise, and inactivity [19,20]. Exemplifying this, previous studies have highlighted a positive role for adjuvant LEU for the regulation of MPS (e.g., [21,22,23,24,25]); however, others have demonstrated no beneficial effect of additive LEU in a mixed EAA/protein solution [26,27] or following free LEU supplementation aiming to enhance lean mass [28,29] in a more chronic setting. Despite this, peak plasma LEU concentrations and postprandial rates of MPS have been correlated in cohorts of young [30,31] and older men [32,33], which to our knowledge, is undetermined in women.

Although BLG, specifically, has a high LEU content, a “ceiling effect” may exist regarding LEU availability and stimulation of MPS, as evidenced by a plateau in the dose–response of MPS to ingested WP in young healthy men [30,31]. Research efforts have, therefore, been focused on investigating low-protein doses that contain higher amounts of LEU, aiming to maximise MPS in individuals who, e.g., are unable to consume the recommended daily allowance (RDA) of protein in order to preserve lean muscle mass, for various reasons. Subsequently, previous research has highlighted that suboptimal doses of protein or EAA mixes enriched with LEU can enhance MPS and provide a similar anabolic response to larger doses (e.g., 40 g) of WP in both younger [22,23] and older individuals [19,24,25]. As the impact of BLG, compared to other protein sources, has not been determined, we investigated the efficacy of moderate-dose (~10 g protein) BLG supplementation, compared to an isonitrogenous whey protein isolate (WPI, ~10 g protein), on plasma concentrations of essential amino acids (EAAs), branched-chain amino acids (BCAAs), leucine (LEU), plasma insulin levels, and skeletal muscle myofibrillar fractional synthetic rate (MPS), in rested and [unilateral] exercised conditions, in young healthy men.

## 2. Methodology

### 2.1. Ethical Approval

This study was approved by the University of Nottingham Faculty of Medicine and Health Sciences Research Ethics Committee (reference number: FMHS 207-0221, 12 March 2021), registered at clinicaltrials.gov (registration number: NCT05701202, registered 8 April 2021 (https://www.clinicaltrials.gov/study/NCT05701202?term=NCT05701202&rank=1, accessed on 26 October 2025)) and was conducted in accordance with the Declaration of Helsinki. Written informed consent was obtained from all study participants.

### 2.2. Participant Characteristics and Screening

Ten young male participants (26 ± 2 years; 179 ± 2 cm; 81 ± 3 kg) were recruited via word of mouth, social media adverts on research group pages, and adverts in the local community. After receiving a detailed information sheet outlining the research study, interested participants attended a screening session to assess their eligibility. This session included a previous medical history discussion, measures of height, weight and blood pressure, and an electrocardiogram. Participant eligibility was confirmed by a clinician after assessing all screening results against pre-determined exclusion criteria (BMI < 18 or >35 kg/m^2^, active cardiovascular, cerebrovascular, or respiratory disease, any metabolic disease or malignancy, clotting dysfunction, a history of or current neurological or musculoskeletal conditions, lactose intolerance). Finally, a unilateral knee extension 1-RM assessment was conducted at this session to measure the strength of the dominant leg (Leisure Lines LTD, Leicestershire, UK, Iso Lever) for the determination of acute resistance exercise (RE) intensity on the assessment days. All screening and study sessions were conducted at the University of Nottingham Medical School at Derby, UK.

### 2.3. Experimental Protocol

Participants were randomly assigned (via www.sealedenvelope.com) to consume BLG or WPI (~10 g protein) on their first assessment day, with the other supplement consumed on their second. Each visit was separated by a washout period of 21 to 42 days (period for pragmatic reasons accounting for illness, holidays, commitments, etc.), although almost all participants completed their second visit within 4 weeks. Prior to completion of their first assessment visit, participants were asked to complete a four-day diet diary recording all food and drink consumed. Nutritics Food Management Software (University Education version, https://www.nutritics.com/en/, accessed from 1 September 2022) was used to determine dietary protein intake. Participants were requested not to alter their habitual dietary intake for the duration of the study (i.e., this was not controlled) and were blinded to which protein they consumed on each visit.

**Figure 1:**
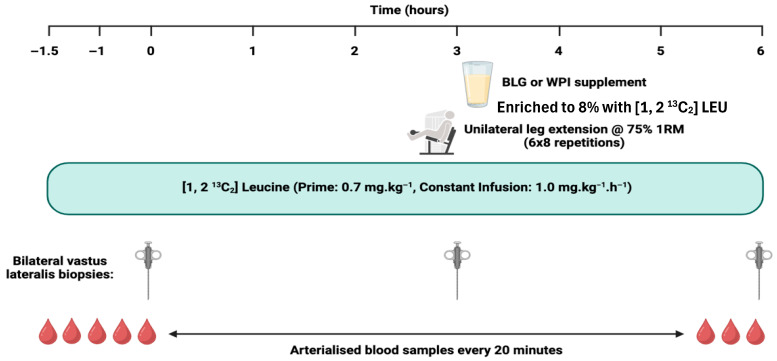
Schematic representation of the study assessment days. Abbreviations: BLG, β-lactoglobulin; WPI, whey protein isolate; 1RM, 1 repetition maximum.

On each assessment day, participants reported to the laboratory at 0800 h, having undergone an overnight fast (≥10 h, water ad libitum) and refrained from intense exercise for at least 72 h. Initially, two venous cannulae were inserted; one was placed into the antecubital fossa for [1,2 ^13^C_2_] LEU stable isotope tracer infusion (99 Atoms % of [1, 2 ^13^C_2_] LEU, Cambridge Isotopes Limited, Cambridge, MA, USA), and one was placed retrograde into a contralateral dorsal vein of the opposite hand for blood sampling. This hand was placed in a heated box at ~55 °C to create an arteriovenous shunt of blood by vasodilation of veins in the fingers, allowing arterialised-venous blood sampling [34]. Blood samples were collected at baseline and every 20 min thereafter until the conclusion of the assessment visit. Plasma samples were collected for the quantification of AA and insulin, and α-ketoisocaproate (α-KIC) enrichment. Following baseline blood samples, a primed, continuous infusion (0.7 mg·g^−1^, 1.0 mg·kg^−1^·h^−1^) of [1, 2 ^13^C_2_] LEU tracer was initiated and continued for the duration of the assessment visit over 7.5 h. At timepoints 0 (i.e., following 1.5 h of tracer infusion), 3, and 6 h after induction of local anaesthetic (~5 mL 1% lignocaine), bilateral skeletal muscle (SKM) biopsies were collected from the *vastus lateralis* muscle (~100–200 mg of tissue) under sterile conditions using a standard conchotome technique. The muscle tissue was washed with ice-cold phosphate-buffered saline to remove excess blood, dissected free of visible fat and connective tissue, snap frozen in liquid nitrogen, and stored at −80 °C until further analysis. SKM biopsies at timepoints 0 and 3 h allowed for postabsorptive MPS to be quantified, whereas biopsies at 3 and 6 h allowed for quantification of postprandial MPS in the fed, rested state (FED) and the fed, exercised (FED-EX) state. Approximately 20 min before the second set of biopsies (i.e., at 3 h), participants performed a bout of unilateral RE (leg extension; 6 sets of 8 repetitions at 75% 1RM with a 2-minute inter-set rest period on the dominant leg (Iso Lever, Leisure Lines LTD)). Immediately after the second set of biopsies, participants ingested the protein feed that they were randomly assigned (Figure 1). Each protein supplement was dissolved in ~250 mL water and consumed as a bolus. To minimise perturbations in plasma isotopic enrichment, beverages were enriched to 8% with [1, 2 ^13^C_2_] LEU tracer. The AA composition of the protein supplements is outlined in Table 1.

### 2.4. Sample Analysis

#### 2.4.1. Plasma Insulin

Plasma insulin concentrations were measured on high-sensitivity human insulin enzyme-linked immunosorbent (ELISA) assays (Mercodia AB, Uppsala, Sweden).

#### 2.4.2. Plasma EAA Concentrations and α-KIC Enrichment

For plasma AA analyses, samples were prepared as previously described [25]. Briefly, 10 µL of a mix of stable isotopically labelled internal standards was added to 100 µL of plasma, treated with urease, and then deproteinised with 0.5 mL ice-cold ethanol at −20 °C for 20 min. Following centrifugation (17,000× *g* for 5 min at 4 °C), the supernatant was decanted and evaporated under nitrogen to dryness. The quinoxalinol KIC derivative was then formed by the addition of o-phenylenediamine solution in HCl and extracted with 2 mL of ethyl acetate. Both solvent (containing quinoxalinol KIC) and aqueous (containing AA) layers were evaporated to dryness and derivatised to their t-butyldimethylsilyl (tBDMS) esters. AA concentrations were quantified against a standard curve of known concentrations alongside α-KIC enrichment using gas chromatography/mass spectrometry (GC/MS; Trace 1300-ISQ, Thermo Scientific, Hemel Hempstead, UK).

#### 2.4.3. Myofibrillar Fractional Synthetic Rate

Myofibrillar proteins were isolated, hydrolysed, and derivatised using our standard techniques [17,25]. Briefly, 20–30 mg of muscle biopsy tissue was homogenised in ice-cold homogenization buffer (50 mM TriseHCL (pH 7.4), 50 mM NaF, 10 mM ß-glycerophosphate disodium salt, 1 mM EDTA, 1 mM EGTA, 1 mM activated Na_3_VO_4_ [all Sigma-Aldrich, Poole, UK]) and a complete protease inhibitor cocktail tablet (Roche, West Sussex, UK) at 10 µL·µg^−1^ of tissue. Following centrifugation at 13,000× *g* for 15 min at 4 °C, the resulting insoluble pellet was washed three times with homogenization buffer to remove excess free AA and solubilised in 0.3 M NaOH to aid separation of the soluble myofibrillar fraction from the insoluble collagen fraction by subsequent centrifugation. The soluble myofibrillar fraction was then precipitated using 1 M PCA, pelleted by centrifugation, and washed twice with 70% ethanol. The protein-bound AA become released by acid hydrolysis using 1 mL 0.1 M HCL and 1 mL of Dowex ion-exchange resin (50W-X8-200) overnight at 110 °C. The free AA were purified and derivatised, and the fractional synthetic rate (FSR) of the myofibrillar proteins was calculated using the precursor–product equation below:

$$\mathrm{FSR}\;(\%/h)\;=\;\left[\frac{\Delta E_{m}}{E_{P}\cdot t}\right]\;\times \;100$$

where Δ*E_m_* is the change in enrichment of bound [1,2 ^13^C_2_] LEU in two sequential biopsies, *t* is the time interval between two biopsies in hours, and *E_P_* is the plasma α-KIC enrichment (a surrogate precursor of the LEU intramuscular pool).

### 2.5. Statistical Analysis

Sample size was based on the primary endpoint of acute changes in MPS in response to nutrition and nutrition-plus-exercise. Based on previous data from our lab investigating feeding responses to a range of protein intakes, using a pooled SD of acute MPS measures of 0.022, it was determined that a sample size of 8 in each group would be able to detect a difference in fasted to fed MPS >30% at >80% power (based on a Cohen’s d estimation of effect size of 1.4) with a 0.05 two-sided significance.

Data was checked for normal distribution using a Shapiro–Wilk test. Myofibrillar FSR (i.e., MPS) and plasma insulin concentrations were analysed using two-way repeated measures ANOVA (supplement × time) with multiple comparisons analysis using Sidak’s correction. Plasma AA concentrations were analysed via mixed-effects analysis (supplement × time) with multiple comparisons analysis using Sidak’s correction. Integrated area under the curve above baseline (iAUC) analysis was determined for plasma EAA, BCAA, and LEU concentrations and analysed via Student’s paired *t*-test. All analyses were performed in GraphPad Prism version 9 (GraphPad Software Inc, San Diego, CA, USA). The alpha level of significance was set at *p* < 0.05. Data is presented as mean ± SEM unless stated otherwise.

## 3. Results

### 3.1. Plasma Leucine, BCAA, and EAA Concentrations

**Figure 2:**
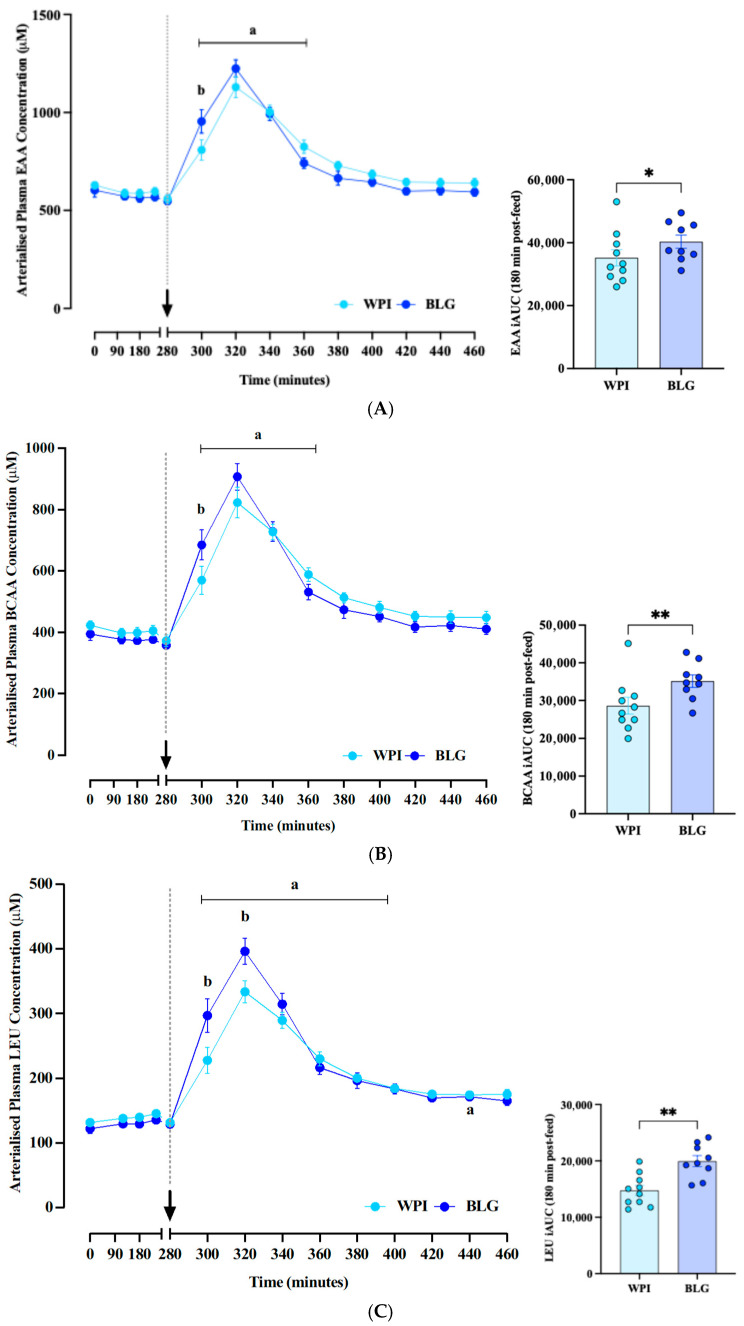
Effects of whey protein isolate (WPI; *n* = 10) and β-lactoglobulin (BLG; *n* = 9) on arterialised plasma essential amino acid (EAA, (**A**)); branched-chain amino acid (BCAA, (**B**)); and leucine (LEU, (**C**)) concentrations. The arrow and dotted line indicate the consumption of protein supplements. Data are presented as mean ± SEM. a: significant difference vs. basal (* *p* < 0.05; ** *p* < 0.01); b: significant difference between groups (*p* < 0.05).

Please note the variable subject *n* in the figures due to sample attrition. Arterialised plasma LEU (main time effect: *p* < 0.0001; time × supplement effect: *p* < 0.0001; Figure 2A) and BCAA (main time effect: *p* < 0.0001; time × supplement effect: *p* > 0.002; Figure 2B) concentrations increased rapidly in both WPI and BLG conditions, peaking at ~40 min following feeding, before returning to baseline at 100–120 min post-feed. With BLG, plasma LEU and BCAA concentrations rose significantly more than the WPI in the first 40 min post-feed, with peak leucinaemia also being greater (396 ± 20 μM vs. 334 ± 17 μM; *p* = 0.0009). In terms of AUC analyses, LEU AUC was significantly different between the WPI and BLG groups (14,762 ± 2785 μM·min vs. 19,954 ± 2970 μM·min, *p* = 0.007). Similarly, BCAA AUC was significantly different between WPI and BLG groups (28,654 ± 6970 μM·min vs. 35,132 ± 4962 μM·min, *p* = 0.001). EAA AUC was also significantly different between WPI and BLG groups (35,210 ± 8142 μM·min vs. 40,321 ± 6275 μM·min, *p* = 0.023) with BLG showing superior aminoacidaemia across the spectrum of EAA clusters.

### 3.2. Plasma Insulin Concentrations

**Figure 3:**
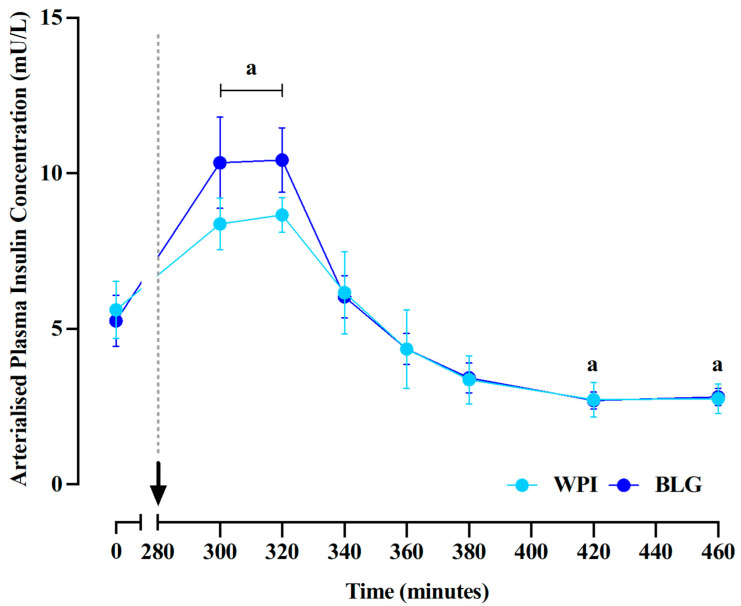
Plasma insulin concentrations (*n* = 10). Data are presented as mean ± SEM. a: significant difference vs. basal (*p* < 0.05).

Plasma insulin concentrations (main time effect: *p* < 0.0001; Figure 2C) increased above postabsorptive levels, peaking at 8.7 ± 1.8 mU/L and 10.4 ± 1.0 mU/L for WPI and BLG conditions, respectively. For WPI and BLG, plasma insulin concentrations became lower than baseline values at 140 and 180 min following feeding (both *p* = 0.02). No differences in plasma insulin concentrations were observed between supplements at any timepoint or for iAUC. The arrow and dotted line indicate the consumption of protein supplements (Figure 3).

### 3.3. Muscle Protein Synthesis

**Figure 4:**
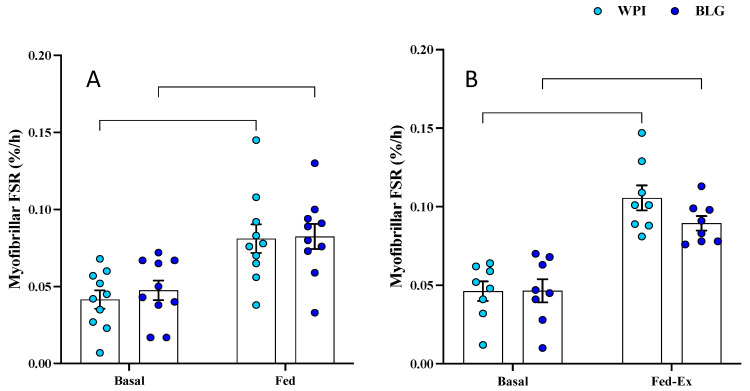
The effects of whey protein isolate (WPI) and β-lactoglobulin (BLG) on skeletal muscle myofibrillar fractional synthetic rate in response to feeding (FED; *n* = 10 (**A**) and feeding-plus-acute resistance exercise (FED-EX; *n* = 8); (**B**). Data presented as mean (bars) ± SEM, with individual data points overlaid.

Baseline rates of myofibrillar FSR (i.e., MPS) did not differ between legs (i.e., fasted vs. fasted-ex leg) or across conditions (i.e., fasted/fasted-ex legs for WPI vs. BLG groups). FED MPS rates increased (main time effect: *p* < 0.0001; Figure 4A) in response to both WPI (0.042 ± 0.006%/h vs. 0.081 ± 0.009%/h; *p* = 0.0006) and BLG (0.048 ± 0.006%/h vs. 0.083 ± 0.008%/h; *p* = 0.0019), with no difference in response between supplements. Likewise, FED-EX MPS rates (*n* = 8; main time effect: *p* < 0.0001; Figure 4B) increased following both WPI (0.046 ± 0.006%/h vs. 0.106 ± 0.008%/h; *p* < 0.0001) and BLG (0.047 ± 0.007%/h vs. 0.090 ± 0.005%/h; *p* = 0.0008), with no difference between supplements.

## 4. Discussion

The use of LEU-enriched EAA/WP has gained traction over recent years as an alternative means to maximise MPS at lower, suboptimal protein doses [17,19,24,35]. Such research studies often involved the addition of free LEU to existing WP and/or EAA mixtures, rather than adopting a strategy of natural enrichment. As such, we compared the effects of a novel WP isolate with a naturally superior LEU content, BLG, to an isonitrogenous WPI at a ‘suboptimal’ dose of protein (10 g), on the effects of muscle anabolism in FED and FED-EX states in young healthy males.

Regarding plasma observations, we noted EAA, BCAA, and LEU concentrations to significantly increase following feeding of both BLG and WPI. In both supplements, we observed peak plasma EAA, BCAA, and LEU concentrations to occur at ~40 min following feeding. BLG notably achieved greater peak and AUC leucinemia, with EAA, BCAA, and LEU concentrations also exhibiting a greater rise over the first 20 min post-feed. This mirrors results observed in previous research studies with supplements containing greater LEU content (e.g., [22,24,25] and in previous studies investigating BLG [14,36]. BLG and WPI also both elicited a predictable insulinotropic response to feeding, with similar increases in plasma insulin following feeding, in agreement with a similar study wherein larger protein supplements (25 g BLG or WPI) were given [36].

In terms of MPS, BLG, at a dose deemed suboptimal for maximal muscle anabolism [30,31,37], significantly increased rates of MPS in both the FED and FED-EX states, with no group differences being observed. Previous research demonstrated that ~10 g EAA in non-exercised conditions [30], or ~8.6 g EAA in exercised conditions [38], was sufficient to stimulate MPS robustly and maximally in healthy young participants. In the current study, both BLG and WPI had lower quantities of EAA than thought optimal (5.6 g vs. 4.7 g EAA, respectively), yet robust stimulation of MPS was still achieved. We also note that the LEU content of BLG (~1.57 g) and WPI (~1.02 g) was quite different; however, MPS was indistinguishable between BLG and WPI (at least in young males where anabolic resistance is less likely). Although BLG contained ~50% more LEU, the difference in bioavailable LEU was not as large, perhaps reflecting splanchnic utilisation (or faster systemic clearance), which minimised any potential dose effect of increased LEU. Similarly to these findings, previous studies have demonstrated that a protein dose of 6.3 g WP (~0.75 g LEU) robustly stimulated fed-state MPS over 3 h in young individuals, comparable to 25 g of whey [22]. Furthermore, in a study by Katsanos et al., young individuals showed a similar MPS response when consuming 6.7 g EAA containing either 26% LEU (1.7 g) or 41% LEU (2.8 g) [21]. Therefore, we contend that in healthy young men, the LEU content provided by ~10 g of either BLG (1.57 g) or WPI (1.02 g) appears to be sufficient to robustly stimulate MPS similarly when adequate amounts of the other EAA are provided. Notably, other EAA than LEU also stimulate MPS, which previous classical studies demonstrated when giving a flooding dose of either valine, phenylalanine, or threonine individually to humans [39].

With research utilising LEU-enriched protein supplementation at lower than optimal doses, questions have arisen as to whether a true dose–response to protein feeding exists if maximal MPS stimulation can be achieved by other means, i.e., LEU-enriched EAA. Indeed, even though suboptimal protein doses with enriched LEU have elicited comparable MPS responses compared to larger doses of protein, this is often only in the early phase (~90–120 min) of MPS stimulation—particularly in the absence of exercise [22,24,25]. In some circumstances, larger doses of WP or LEU supplementation have been evidenced to sustain the increase in or lead to greater stimulation in MPS in young men over longer assessment periods (i.e., ~4–5 h) and where exercise is a variable to nutrition alone [23,25]. Further work utilising a dose of protein per kg body weight or lean body mass is required to fully elucidate the existence of an MPS dose–response to protein or LEU intake. In sum, there is good evidence of a dose–response of MPS to protein, EAA/LEU ingestion [30,31,40,41], and that there is a maximal response to providing an additional AA substrate of around 20 g WP, 10 g EAA, or 3 g LEU in young males, despite stimulation being prolonged by prior exercise [35].

## 5. Conclusions

In conclusion, a “suboptimal” (20 g being suggested optimal) 10 g dose of BLG in young individuals leads to significant increases in plasma EAA, BCAA, and insulin concentrations with greater plasma leucinaemia achieved compared to WPI, in addition to significant stimulation of MPS in the FED and FED-EX states. In terms of limitations, we acknowledge the lack of assessment of the impact of BLG on LEU kinetics and muscle anabolism in females. Moreover, larger sample sizes and greater diversity in age and ethnicity are required. In addition, while taking a pragmatic approach in refinement of BLG and comparing to WPI, it is acknowledged these are not precisely balanced in non-LEU individual EAA content (despite being well-matched for total EAA), which could influence outcomes. Future work should also focus on dose–responses per unit body/muscle mass, clinical populations who may benefit from the superior LEU content due to anabolic resistance to protein nutrition/reduced ability to utilise large amounts of protein and amino acids, and the satiating effect of protein impacting their macronutrient intake.

## Figures and Tables

**Table 1:** Amino acid composition of β-lactoglobulin (BLG) and whey protein isolate (WPI) supplements.

Amino Acid	BLG (g/100 g Protein)	WPI (g/100 g Protein)
Alanine	7.0	5.0
Arginine	2.7	1.8
Asparagine	11.9	10.7
Cysteine	3.0	2.5
Glutamic Acid	20.6	16.8
Glycine	1.4	1.5
Histidine	1.6	1.6
Isoleucine	6.4	6.4
Leucine	15.7 (13.6%)	10.2 (10.4%)
Lysine	12.2	9.2
Methionine	2.8	2.1
Phenylalanine	3.5	2.9
Proline	5.5	6.0
Serine	3.9	4.6
Threonine	5.5	7.1
Tryptophan	2.2	1.8
Tyrosine	3.6	2.7
Valine Total EAA (%)	6.1 115.6 48.4	5.6 98.5 47.6

## Data Availability

The original contributions presented in this study are included in the article. Further inquiries can be directed to the corresponding author.

## Abbreviations

1RM, 1 repetition maximum; AA, amino acid; BLG, β-lactoglobulin; EAA, essential amino acid; FED, feeding-only; FED-EX, feeding-plus-exercise; FSR, fractional synthetic rate; GIP, glucose-dependent insulinotropic polypeptide; iAUC, integrated area under the curve above baseline; LEU, leucine; MPB, muscle protein breakdown; MPS, muscle protein synthesis; NEAA, non-essential amino acids; RDA, recommended daily allowance; RE, resistance exercise; SKM, skeletal muscle; WP, whey protein; WPI, whey protein isolate.
